# One-Year Retrospective Review of Psychiatric Consultations in Lassa Fever, Southern Nigeria

**DOI:** 10.3201/eid2612.200084

**Published:** 2020-12

**Authors:** Esther O. Okogbenin, Michael O. Obagaye, Benjamin E. Aweh, Williams O. Eriyo, Sylvanus A. Okogbenin, Peter O. Okokhere

**Affiliations:** Ambrose Alli University, Ekpoma, Edo State, Nigeria and Institute of Lassa Fever Research and Control, Irrua Specialist Teaching Hospital, Edo State, Nigeria (E.O. Okogbenin, S.A. Okogbenin, P.O. Okokhere);; Institute of Lassa Fever Research and Control, Irrua Specialist Teaching Hospital, Edo State (M.O. Obagaye, B.E. Aweh, W.O. Eriyo)

**Keywords:** psychiatric manifestations, delirium, Lassa fever, psychiatric consultations, Nigeria, viruses

## Abstract

We conducted a retrospective review of psychiatric consultations for hospitalized patients with Lassa fever in southern Nigeria. Ten (8.8%) of 113 patients had psychiatric consultations. Delirium was the most common psychiatric manifestation complicating Lassa fever. Findings suggest that psychiatric intervention could improve overall outcomes of Lassa fever.

Viral hemorrhagic fever viruses may cause a wide spectrum of neurologic manifestations, including psychiatric syndromes (*1**–**3*). To the best of our knowledge, only 1 study, performed in 1991 in Sierra Leone, attempted to show that psychiatric syndromes are possible in acute Lassa fever (LF) (*3*). This case series, reported by Solbrig and McCormick, showed psychiatric syndromes including delirium, depression, and abnormal behavior in 3 of 9 patients with central nervous system (CNS) manifestations (*3*).

This review was conducted at the Institute of Lassa Fever Research and Control, Irrua Specialist Teaching Hospital Irrua, Edo state, Nigeria, a national LF referral center. We retrospectively reviewed the files of patients with Lassa virus reverse transcription PCR–positive blood samples, who were admitted at the center during 2012, and included in the study patients who had psychiatric consultations. A questionnaire designed by the researchers was used to collect information from patient files. We examined each file for psychopathology and coded the eventual diagnosis using the International Classification of Diseases, Tenth Revision (ICD-10) (*4*).

Ten (8.8%) of 113 hospitalized patients with LF had psychiatric consultations. All 10 patients met ICD-10 criteria for delirium (hyperactive motor type) and 2 had co-occurring depression. None of the 10 patients had a history of psychiatric illness. All 10 patients received supportive psychotherapy and haloperidol in low doses (2.5−5 mg daily). Citalopram (20 mg) was used for depression. All 113 patients were given ribavirin and received symptomatic management and treatment of medical complications and other preexisting conditions by the infectious disease physicians. All patients recovered from delirium and depression within 3 weeks and survived the infection despite an overall mortality rate of 45.1% (54/113) in the hospitalized patients (Table).

**Table:** Sociodemographic and clinical characteristics observed in a retrospective review of psychiatric consultations in patients with Lassa fever conducted at the Irrua Specialist Teaching Hospital, Irrua, Edo State, Nigeria, January–December 2012*

ID	Age, y/ sex	Duration of fever, d	Temperature at onset of psychiatric symptoms, °C	Medical complications	Preexisting conditions	Family history of mental illness	ICD-10 diagnosis
1	25/M	14	38.0	Acute renal failure	No	No	Delirium
2	30/F	14	37.2	Acute renal failure	No	No	Delirium
3	31/M	6	38.0	Anemia, hypokalemia	No	No	Delirium
4	33/F	6	36.8	Low platelet levels	No	No	Delirium
5	38/M	14	38.6	Altered liver enzyme levels, hypernatremia	Diabetes	No	Delirium
6	45/F	14	38.0	Uremia	No	No	Delirium
7	55/F	14	36.5	Anemia	Hypertension	Yes	Delirium
8	60/M	6	38.0	Acute renal failure, septicemia	Hypertension	No	Delirium, depression
9	38/M	14	38.5	Anemia	Diabetes	No	Delirium, depression
10	20/F	10	37.6	Acute renal failure, seizures, anemia	No	No	Delirium

*All patients had fever and tested positive for Lassa fever by reverse transcription PCR. All patients survived. ICD-10, International Statistical Classification of Diseases and Related Health Problems, 10^th^ revision; ID, identification.

The finding of delirium in 100% of our patients with psychiatric manifestations is comparable with findings of a study that evaluated psychiatric illness in a typhoid fever cohort in Nigeria, where delirium was reported in 73% of 26/136 patients with psychiatric symptoms (*5*). Although mild and self-limiting confusion occurs in many febrile illnesses, delirium has been reported to be associated with prolonged hospital stay (14–40 days) in patients with infectious diseases; this was statistically significant (p<0.001) when compared with patients without delirium (hospital stay <14 days) in Nigeria (*6*). The strict ICD-10 criteria require symptoms of delirium to be present in each of the following 5 areas: disturbance of consciousness and attention, cognition, psychomotor, emotional, and sleep-wake cycle disturbances (*4*). Using these criteria, we found delirium in our patients, who were all hyperactive, and ruled out mild confusion. Of note is that fever had subsided in 4 of our patients by the time of onset of psychiatric symptoms. No patient with anxiety was seen, and only 2 patients had co-occurring depression. 

The absence of past psychiatric illness in the patients we studied suggests that LF was likely the direct or indirect cause of delirium/depression in these patients. Psychiatric manifestations and viral infections are linked through a complex interaction; in our patients, this interaction could have been a direct cytopathic effect of LF virus on their CNS. Generally, viruses enter the CNS through several pathways, which may include a hematogenous route, directly breaching the blood–brain barrier, or through infected leukocytes, which then infect vascular endothelial cells (*2**,**7*). A case of infection with Lassa virus in cerebrospinal fluid has been reported in a patient with blood samples negative for Lassa virus (*8*). In fact, psychiatric symptoms without neurologic symptoms may be the initial presentation of viral encephalitis (*9*).

All our patients, like some other patients with severe LF, had various medical complications, such as acute renal failure, septicemia, and electrolyte disturbances. These are well documented etiologic factors for delirium (*10*) and could have contributed to delirium in our patients.

All 10 patients recovered from delirium and depression within 3 weeks of intervention and survived the infection despite an overall mortality rate of 45.1% for patients admitted to the hospital with LF. This is irrespective of the presence of poor prognostic factors in these patients and the fact that the same LF case management protocol was applied to all patients admitted to the center. Unfortunately, there were no data on viral load and oxygen saturation for comparison between our patients and other patients with LF who did not receive psychiatric intervention. Although we cannot adequately explain this excellent prognosis, we note that identifying and managing psychiatric complications could contribute to improved LF outcome.

The limitation of this study was that it was retrospective and looked at only those who had psychiatric consultations, which made the sample size small and did not permit causal inferences. A prospective study might have identified more cases and given more room for a comparative study design. Based on our findings, we recommended prospective studies to determine the pattern of psychiatric manifestations in LF and integrating mental healthcare into the management of LF.
